# Disease classification via interpretable machine learning based on multi-center routine coagulation test

**DOI:** 10.3389/fmolb.2026.1788536

**Published:** 2026-03-04

**Authors:** Feng Dong, Yaqiong Zhang, Weibu Chen, Changmin Wang, Lei Zhang, Xiaoling Gao, Xiaoli Zhang, Minghua Jiang, Guobin Xu, Ruichuang Yang, Yutong Hou, Jiandang Ma, Chuanbao Li, Jun Wu

**Affiliations:** 1 Department of Clinical Laboratory, Beijing Jishuitan Hospital, Capital Medical University, Beijing, China; 2 Department of Clinical Laboratory, Taizhou Key Laboratory of Research and Transformation of Extracellular Vesicles, Taizhou Central Hospital (Taizhou University Hospital), Taizhou, Zhejiang, China; 3 Laboratory Department, Shenzhen People’s Hospital, Shenzhen, Guangdong, China; 4 Center of Clinical Laboratory, People’s Hospital of Xinjiang Uygur Autonomous Region, Urumqi, Xinjiang, China; 5 Department of Clinical Laboratory, The Second Affiliated Hospital of Xi’an Jiaotong University, Xi’an, Shaanxi, China; 6 Department of Laboratory Medicine, Hainan General Hospital, Haikou, Hainan, China; 7 The Affiliated Yongchuan Hospital of Chongqing Medical University, Chongqing, China; 8 The Second Affiliated Hospital of Wenzhou Medical University, Wenzhou, Zhejiang, China; 9 Key Laboratory of Carcinogenesis and Translational Research (Ministry of Education), Department of Clinical Laboratory, Peking University Cancer Hospital and Institute, Beijing, China; 10 Shenzhen Mindray Bio-Medical Electronics Co., Ltd., Shenzhen, Guangdong, China; 11 Department of Artificial Intelligence, Beijing Key Lab of Traffic Data Analysis and Mining, School of Computer Science & Technology, Beijing Jiaotong University, Beijing, China; 12 Luoyang Central Hospital, Luoyang, Henan, China; 13 Department of Laboratory Medicine, Beijing Hospital, National Center of Gerontology, Institute of Geriatric Medicine, Chinese Academy of Medical Sciences, Beijing, China

**Keywords:** disease classification, interpretability analysis, machine learning, multi-center coagulation test, SHapley Additive exPlanations

## Abstract

**Background:**

This study aims to establish an interpretable disease classification model via machine learning and identify key features related to the disease to assist clinical disease diagnosis based on a multi-center CX9000 routine coagulation test.

**Methods:**

Data from 11 hospitals were collected. An unsupervised clustering model was used to extract classification patterns, and clinical experts assigned disease labels. Multiple machine learning models, including Random Forest, SVM, Decision Tree, Naive Bayes, MLP, XGBoost, and LightGBM, were trained. Ten-fold cross validation and external validation were performed. For external validation, models were trained with data from 8 hospitals (˜90%) and tested on the remaining 2 hospitals (˜10%). SHAP and Decision Tree analysis were used for interpretability.

**Results:**

Clear clustering patterns were observed for valvular heart disease (VHD) and pulmonary infection (PI). LightGBM achieved the best performance in both tasks. In cross validation, the mean F1-scores were 0.8890 and 0.7233, and the mean AUCs were 0.9500 and 0.8023. External validation showed strong generalization, with mean F1-scores of 0.9259 and 0.7464 and mean AUCs of 0.9493 and 0.8297. The sample visualization by t-SNE and the interpretable analysis by SHAP and Decision Trees identified some key classification features, i.e., international normalized ratio (INR) for VHD and age for PI.

**Conclusion:**

Machine learning models based on multi-center coagulation tests provide effective and interpretable disease classification, supporting clinical diagnostic automation.

## 1 Introduction

The growth of medical data and analytical advances enable data-driven research for personalized medicine and precise diagnosis. By mining patients’ clinical data and combining them with machine learning models, clinicians can more accurately identify disease patterns, predict disease risks, and optimize diagnosis and treatment plans (Haug and Drazen., 2023; Shehab et al. 2022; Shamout et al., 2020). However, real-world clinical data complexity, including high-dimensional heterogeneous distributions and overlapping disease features, presents significant challenges to conventional analytical methods.

In recent years, classification models based on machine learning have shown significant advantages in medical diagnosis. They can not only process high-dimensional nonlinear data, but also reveal the contribution of features to model decisions through explanatory tools. In disease classification tasks, especially the binary classification problem of identifying specific diseases based on indicator information, machine learning methods have been proven to be an efficient and reliable solution. For example, Alatrany et al. (2024) used Support Vector Machine (SVM) for early diagnosis of Alzheimer’s disease, demonstrating the potential of the model in complex disease identification. Chen et al. (2025) used machine learning models such as Logistic Regression, Random Forest, XGBoost, and LightGBM to predict high-risk chest pain. Khan et al. Khan et al. (2023) used various machine learning methods to classify and predict patients with cardiovascular disease. Kadhim and Radhi (2023) and Oktafiani et al. (2024) identified heart disease patients based on models such as SVM, K-nearest neighbor, Decision Tree, and Random Forest. Addisu et al. (2025) combined machine learning methods with transfer learning for the early detection of heart disease. Malakouti. (2023) effectively determined whether a patient had heart disease through machine learning analysis of electrocardiogram data. Li et al. (2020) developed a heart disease diagnosis system based on machine learning while exploring the performance of the model, providing support for practical application scenarios.

This study focuses on multi-center routine coagulation test data from patients, using an unsupervised clustering model to reveal distinct coagulation features in specific indicators for valvular heart disease (VHD) and pulmonary infection (PI). Based on these findings, two binary classification tasks were designed. To accomplish these tasks, various machine learning models, including Random Forest, SVM, Decision Tree, Naive Bayes, multilayer perceptron (MLP), XGBoost, and LightGBM, were utilized for comparative performance analysis. The generalization capability of the models was further validated through external validation experiments using data from multiple hospitals. Additionally, the interpretable framework SHapley Additive exPlanations (SHAP) and Decision Tree analysis module were applied to interpret the results of disease classification and identify key features. These results not only highlight the key features but also demonstrate the potential of machine learning models in enhancing clinical disease diagnosis, providing theoretical insights and practical references for auxiliary diagnosis.

## 2 Materials and methods

### 2.1 Data description

The data used in this experiment were derived from the clinical records of patients from 11 hospitals between May 2023 and May 2024, including their personal and disease-related information.

Personal information includes date, patient index, name, age, gender, status, sampling time, sample integrity, number of tests, and test indicators. The test indicators include activated partial thromboplastin time (APTT), prothrombin time (PT), international normalized ratio (INR), thrombin time (TT), fibrinogen (FIB), fibrin degradation products (FDP), D-dimer (D-D), and antithrombin III activity (AT III), all of which were measured using the CX9000 automatic coagulation analyzer. In the experiment, the patient features used include age and coagulation indicators.

Disease information encompasses disease index, disease name, requesting department, and medication usage. Disease classification was performed using International Classification of Diseases (ICD) codes. After data cleaning and classification, a total of 6,301 samples were obtained, covering 10 diseases: cerebral infarction (CI), cerebrovascular disease (CVD), coronary heart disease (CHD), valvular heart disease (VHD), hypertension (HTN), pulmonary infection (PI), lung cancer (LC), chronic obstructive pulmonary disease (COPD), diabetes mellitus (DM), and type 2 diabetes mellitus (T2DM). The disease index, name, and number of samples are shown in Table 1.

**TABLE 1:** Statistics of the number of diseases.

Index	Disease	Numbers
1	Cerebral infarction (CI)	835
2	Cerebrovascular disease (CVD)	597
3	Coronary heart disease (CHD)	1,479
4	Valvular heart disease (VHD)	427
5	Hypertension (HTN)	807
6	Pulmonary infection (PI)	790
7	Lung cancer (LC)	539
8	Chronic obstructive pulmonary disease (COPD)	183
9	Diabetes mellitus (DM)	300
10	Type 2 diabetes mellitus (T2DM)	344

### 2.2 Disease classification method

In this study, seven classic machine learning models were used to handle disease classification tasks, including SVM, Decision Tree, Random Forest, Naive Bayes, MLP, XGBoost, and LightGBM. These models each have different advantages and characteristics and can mine disease patterns from coagulation data.

The goal of SVM Cortes and Vapnik (1995) is to improve the generalization ability of classification by finding an optimal hyperplane that maximizes the interval between two types of samples. In the case of linear separability, SVM constructs the optimal hyperplane by optimizing the constraint problem as shown in Equation 1:

$$\min_{w,b}\frac{1}{2}{\left\|w\right\|}^{2}\;s.t.\;y_{i}\left(w^{T}x_{i}+b\right)\geq 1,\forall i,$$ (1)

where $x_{i}$ represents the feature vector of the i -th sample, $y_{i}$ is the label of the i -th patient, indicating the patient’s disease category, w is the normal vector of the hyperplane, and b is the displacement term.

When the data is linearly inseparable, SVM introduces soft margins and slack variables, and balances misclassification with margin maximization by adding a penalty term. To solve the problem of nonlinear classification, SVM uses kernel techniques to map data to a high-dimensional feature space and achieves nonlinear classification by finding a hyperplane in this space. Common kernel functions include linear kernels, Gaussian kernels, and polynomial kernels.

The core idea of the Decision Tree Quinlan, (1986) is to divide the samples into more homogeneous subsets by recursively splitting the feature space, thereby building an easy-to-interpret tree model. The key to building a Decision Tree is to select the optimal split point. For classification tasks, the Gini Impurity is used as the splitting indicator by default, which is defined as Equation 2:

$$G\left(f\right)=1-\overset{C}{\underset{c=1}{\sum }}p_{c}^{2},$$ (2)

where $p_{c}$ represents the proportion of samples in category c . By minimizing the Gini Impurity of the split subset, the Decision Tree achieves the best division of samples.

Random Forest Breiman (2001) is an algorithm based on ensemble learning, which consists of multiple Decision Trees. It enhances the generalization ability of the model through “sample randomness” and “feature randomness”. During the training process, Random Forest randomly extracts subsets from the original data set through bootstrap sampling, and trains a Decision Tree for each subset. To further increase diversity, each tree randomly selects some features for optimal splitting when splitting a node. This mechanism reduces the dependence of a single tree on specific samples or features and reduces the risk of overfitting. Finally, the results are integrated through majority voting, and the final prediction output is shown in Equation 3:

$${\hat{y}}_{j}=arg\max_{c\in \left\{0,1\right\}}\overset{k}{\underset{i=1}{\sum }}{\hat{y}}_{i}^{\left(j\right)}.$$ (3)

Naive Bayes Duda and Hart (1973) is a probabilistic classification method based on Bayes’ theorem, which assumes conditional independence between features. For a given sample x , Naive Bayes predicts its category y by maximizing the posterior probability P(y|x) according to Equation 4:

$$P\left(y|x\right)\propto P\left(y\right)\overset{n}{\underset{i=1}{\prod }}P\left(x_{i}|y\right),$$ (4)

where P(y) is the prior probability, $P\left(x_{i}|y\right)$ is the conditional probability, which is usually estimated through sample statistics.

MLP Rumelhart et al. (1986) is composed of input, multiple hidden, and output layers, employing fully connected structures and activation functions for nonlinear transformations. The core of MLP is to minimize the loss function through the backpropagation algorithm. Specifically, let the input sample be x , the weight matrix be W , and the activation function be f(⋅) , then the output of the hidden layer is obtained using Equation 5:

$$h=f\left(W^{t}x+b\right).$$ (5)

The final prediction result is calculated by the output layer, and then the loss function is used to calculate the loss between the target value and the output layer. The weights are iteratively updated through the gradient descent method to reduce the prediction error.

XGBoost Chen, (2016) is an efficient ensemble learning algorithm built on Gradient Boosted Decision Trees (GBDT) that aims to improve model accuracy and training speed through parallel computation and algorithmic optimizations. Unlike traditional GBDT, which only uses first-order derivative information, XGBoost applies a second-order Taylor expansion to the loss function and introduces a regularization term to control model complexity and thus mitigate overfitting. At the tth iteration its objective is approximated as Equation 6:

$$Obj^{\left(t\right)}\approx \overset{n}{\underset{i=1}{\sum }}\left[g_{i}f_{t}\left(x_{i}\right)+\frac{1}{2}h_{i}f_{t}^{2}\left(x_{i}\right)\right]+\Omega \left(f_{t}\right),$$ (6)

where $g_{i}$ and $h_{i}$ denote the first and second derivatives of the loss with respect to the previous prediction, and $\omega \left(f_{t}\right)$ is the regularizer that includes the number of leaves and the L2 norm of leaf weights. By minimizing this objective, XGBoost can precisely compute optimal leaf weights, enabling deep mining and classification of features in coagulation data.

LightGBM Ke et al. (2017) is a lightweight and efficient gradient boosting framework optimized for large-scale data and high-dimensional features. To address the computational cost of finding optimal split points in traditional GBDT, LightGBM employs a histogram based Decision Tree algorithm that discretizes continuous features into k bins, greatly reducing memory usage and accelerating computation. In terms of tree growth strategy, LightGBM departs from the conventional level-wise growth and adopts a growth strategy that splits the leaf with the largest gain subject to a depth limit, expressed as Equation 7:

$${\left(Leaf\right)}^{*}=argmax_{\Delta \in Leaves}\left(Gain\left(\Delta \right)\right).$$ (7)

This strategy selects at each step the leaf whose split yields the maximum Gain, which for the same number of splits can produce lower error than the level-wise growth strategy.

### 2.3 Machine learning interpretability framework SHAP

SHAP Lundberg (2017) is a model explanation method based on game theory, which is used to quantify the contribution of each feature to the prediction results of the machine learning model. This method has significant advantages in interpretability and theoretical rigor, and has become an important tool in model interpretability research.

SHAP is based on the idea of Shapley value, treating features as “players” in a cooperative game, and the model’s prediction results as “profits”. By calculating its marginal contribution to model prediction under different feature combinations, SHAP assigns a contribution value to each feature using Equation 8:

$$\begin{array}{c} \phi_{i}=\underset{S\in X\backslash \left\{i\right\}}{\sum }\frac{|S|!\left(|X|-|S|-1\right)!}{|X|!}\left[f\left(S\cup \left\{i\right\}\right)-f\left(S\right)\right], \end{array}$$ (8)

where $\phi_{i}$ is the Shapley value of feature i , X is the set of all features, S is a subset of features, and f(S) is the predicted value of the model trained using only the feature subset S .

SHAP value satisfies local accuracy, consistency and feature irrelevance, can take into account the marginal contribution of features in all possible subsets, comprehensively evaluate their global importance, and help researchers deeply understand the model decision-making mechanism.

### 2.4 Experimental settings

#### 2.4.1 Dataset

We use the data described in Section 2.1 to construct datasets for the VHD classification task and the PI classification task. When constructing the dataset, we first set the label of the target disease to 1 as a positive sample, and then randomly select the same number of samples from other diseases and set the labels of these samples to 0 as negative samples. The training process uses 10-fold cross validation, that is, the dataset is divided into ten parts, and 9 of them are used as training data and 1 as test data in turn for experiments.

#### 2.4.2 Evaluation metrics

The evaluation indicators of the experiment are accuracy, recall, F1-score and AUC. The model is trained by 10-fold cross validation, so the results are the average of 10 experiments, and the calculations of accuracy, recall and F1-Score of each experiment are defined in Equations 9–12:

$$Accuracy=\frac{TP+TN}{TP+TN+FP+FN},$$ (9)

$$Recall=\frac{TP}{TP+FN},$$ (10)

$$F1-score=\frac{2\cdot Precision\cdot Recall}{Precision+Recall},$$ (11)

$$Precision=\frac{TP}{TP+FP},$$ (12)

where TP represents the number of true positive examples, TN represents the number of true negative examples, FP represents the number of false positive examples, and FN represents the number of false negative examples.

AUC refers to the area under the receiver operating characteristic (ROC) curve, which measures the ability of the model to distinguish between positive and negative samples. The value range of AUC is [0, 1], and the closer it is to 1, the better the classification performance.

#### 2.4.3 Hyperparameter settings

All experiments were implemented in Python on an i7-10875H CPU, with a fixed seed value of 42 to ensure reproducibility. Hyperparameters were optimized via grid search, and the detailed settings for each model are presented in Table 2. Specifically, SVM uses a linear kernel as the kernel function. The Decision Tree has a maximum depth of 10, a minimum of 50 samples required to split an internal node, and a minimum of 40 samples required at a leaf node. The Random Forest contains 10 Decision Trees, and its maximum depth, minimum samples required before node splitting, and minimum samples required at leaf nodes follow the same settings as the Decision Tree. The MLP has one hidden layer with 100 neurons, uses Adam Kingma (2014) as the optimizer, adopts a learning rate of 0.001, and sets the maximum number of iterations to 800. The XGBoost model uses gbtree as the booster, with 100 estimators, a maximum tree depth of 6, a learning rate of 0.3, and log loss as the evaluation metric. The LightGBM model employs the gbdt boosting strategy, with 100 estimators, a maximum number of 31 leaf nodes, and a learning rate of 0.1.

**TABLE 2:** Hyperparameter settings for each model.

Classification models	Hyperparameters	Value
SVM	Kernel	Linear
Decision tree	max_depth	10
	min_samples_split	50
	min_samples_leaf	40
Random forest	n_estimators	15
	max_depth	10
	min_samples_split	50
	min_samples_leaf	40
MLP	hidden_layer_sizes	100
	Activation	relu
	Solver	Adam
	learning_rate_init	0.001
	max_iter	800
XGBoost	Booster	Gbtree
	n_estimators	100
	max_depth	6
	learning_rate	0.3
	eval_metric	Logloss
LightGBM	boosting_type	gbdt
	n_estimators	100
	num_leaves	31
	learning_rate	0.1

## 3 Experimental results and analysis

### 3.1 Data analysis and disease selection analysis

**FIGURE 1:**
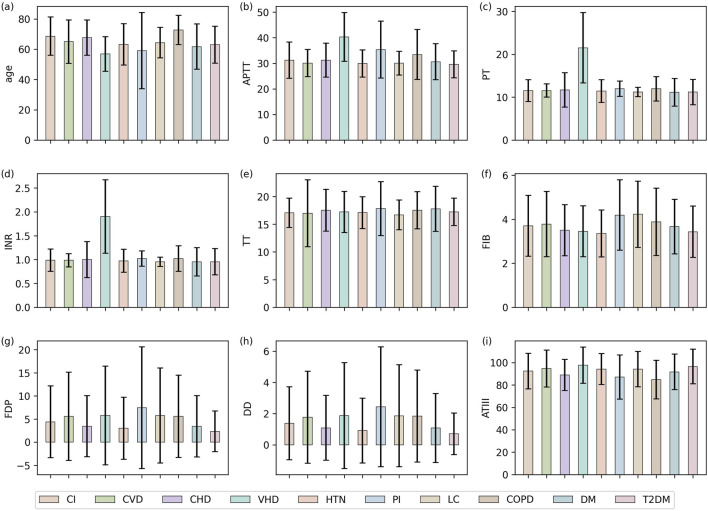
Mean and variance distribution of each feature in different diseases, where **(a–i)** show the mean and variance of age, APTT, PT, INR, TT, FIB, FDP, DD, and ATIII, respectively.

Before conducting the experiment, we generated error bar charts of 9 features across 10 patient categories, as shown in Figure 1. While there was no obvious difference in the characteristics of most diseases, some could still be distinguished by these indicators. For example, the PT and INR of VHD were significantly higher than other diseases, and the FDP and D-D of PI were the highest. In VHD, mechanical hemodynamic changes chronically activate the extrinsic coagulation pathway, while the frequent clinical use of long-term anticoagulant therapy further significantly elevates PT and INR levels. In contrast, the coagulation changes in PI are mainly mediated by the systemic inflammatory response, which induces a hypercoagulable state characterized by elevated FIB and increased D-D and FDP due to secondary fibrinolysis. These different mechanisms provide a robust biological basis for using low-cost, accessible routine coagulation indicators as effective tools for differential diagnosis. Therefore, we selected these two diseases as target diseases to construct the disease classification task.

**FIGURE 2:**
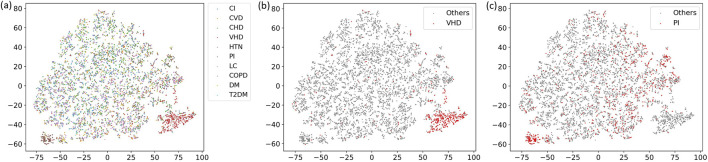
t-SNE visualization analysis. **(a)** shows all disease categories, **(b)** highlights the VHD group, and **(c)** highlights the PI group.

Then, t-SNE was used to visualize the patient features in the dataset, and the results are shown in Figure 2. Figure 2a uses 10 different colors to distinguish patients with different diseases. It could be seen that most diseases were relatively scattered, indicating a certain degree of overlap between patient features and low disease differentiation. However, some diseases formed relatively independent clusters, such as the red cluster in the lower right and the brown cluster in the lower left. These two types of diseases were highlighted, and Figures 2b,c were obtained. Figure 2b marks patients with VHD in red. Patients with VHD were concentrated in the lower right area, demonstrating a good clustering effect. Figure 2c marks patients with PI. The characteristics of PI were less clustered. Some patients were distributed in the areas of patients with other diseases, but they were mainly distributed in the lower left area, showing a certain clustering trend. Therefore, selecting these two diseases as targets was justified.

### 3.2 Cross validation results on disease classification

**FIGURE 3:**
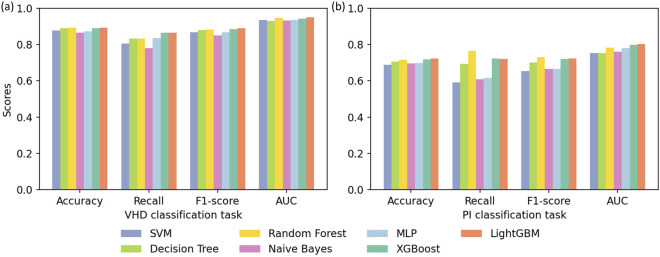
Results of cross validation experiments for **(a)** the VHD classification task and **(b)** the PI classification task.

**FIGURE 4:**
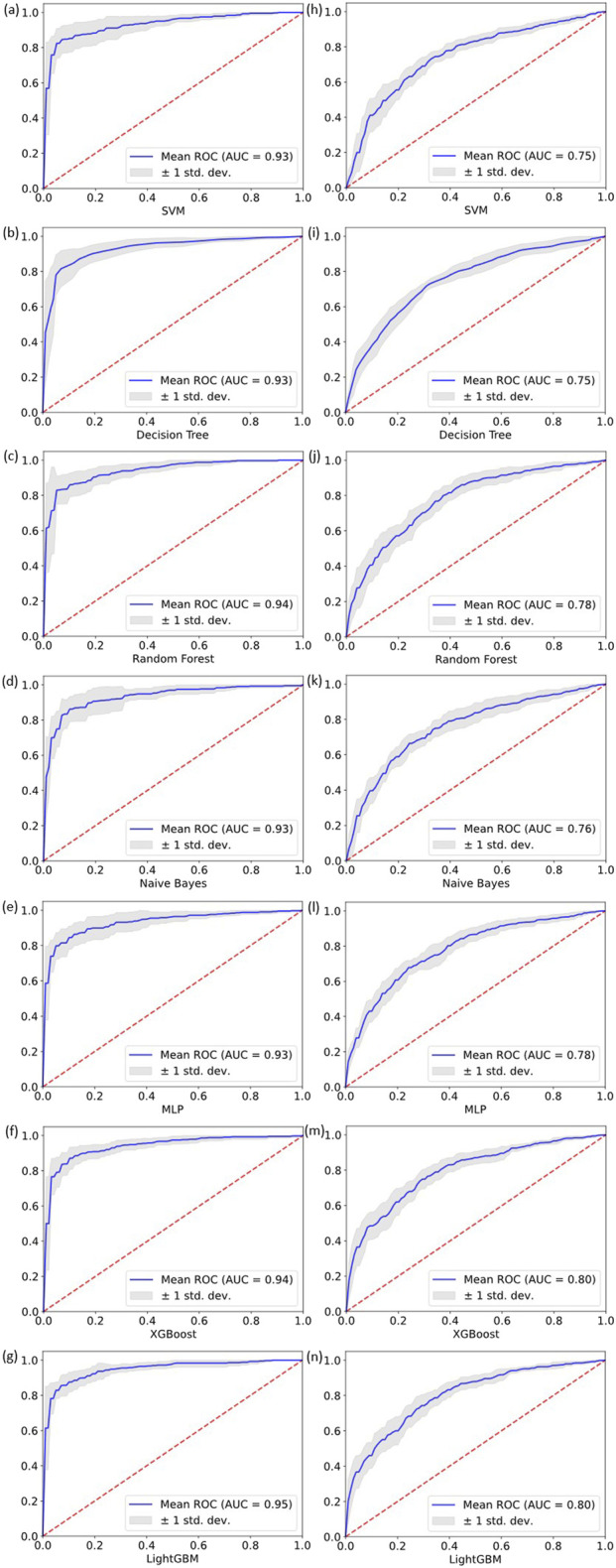
ROC curves for cross validation experiments on two classification tasks. **(a–g)** show the performance of the seven models on the VHD classification task. **(h–n)** show the performance of the seven models on the PI classification task.

The performance of each baseline model on the two disease classification tasks is shown in Figure 3, and the ROC curves of each model are shown in Figure 4. Overall, ensemble learning tree models, including LightGBM, XGBoost and Random Forest, performed markedly better than the other models, and LightGBM achieved the best overall performance.

In particular, LightGBM demonstrated the strongest overall capability in both tasks. It ranked first on all metrics in the VHD task and maintained the highest accuracy (0.7241) and AUC (0.8023) in the PI task. XGBoost followed and showed better overall performance than Random Forest, while Random Forest still retained an advantage in identifying positive samples in the PI task through the highest average recall (0.7646) and the highest F1-score (0.7292). Models including SVM, Decision Tree, MLP and Naive Bayes showed clearly weaker performance than the three ensemble learning models above.

LightGBM and XGBoost, as representative gradient boosting algorithms, train models iteratively to correct the errors of previous predictions, which enables them to capture nonlinear relationships and complex features in the data more deeply and explains their advantage over Random Forest in accuracy and AUC. LightGBM in particular exhibits stronger robustness when handling medical data because it uses an efficient histogram algorithm and a leaf growth strategy. Random Forest relies on a bagging strategy and class weight adjustment, and although it is slightly weaker in the VHD task, its high recall in the PI task indicates strong tolerance to noise.

In contrast, the Decision Tree as a single classifier is less effective than ensemble models when dealing with noise and correlated features. The MLP struggles to converge to an optimal solution when the feature distribution is complex and the data size is limited. The SVM, although able to address some nonlinear patterns through kernel functions, performed poorly on the PI task because it has difficulty modeling highly complex feature relationships. The Naive Bayes model is constrained by its assumption of feature independence and performs poorly when applied to medical data with strong feature correlations.

**FIGURE 5:**
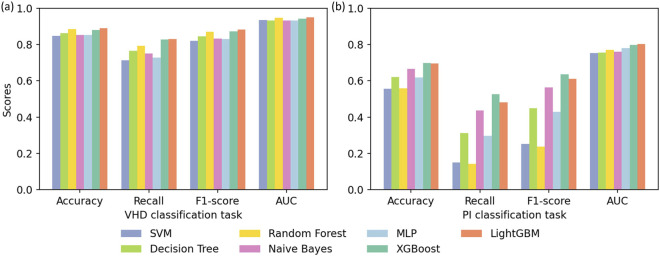
Results of cross validation experiments under the 80% threshold setting for **(a)** the VHD classification task and **(b)** the PI classification task.

To further evaluate model performance in clinical scenarios that require high specificity, we increased the classification threshold to 0.8, meaning that a sample is predicted as positive only when the estimated probability of disease is at least 80%. Figure 5 reports the performance of each model under this strict threshold.

In the VHD classification task, although the threshold was substantially increased, ensemble models such as LightGBM, XGBoost and Random Forest maintained very strong stability. LightGBM achieved an average accuracy of 0.8900 and an F1-score of 0.8824 under this high threshold, again demonstrating its clear advantage on this task. This indicates that the positive and negative samples in this task exhibit relatively distinct characteristics, and the model generally assigns high confidence scores to positive predictions, making it suitable for strict clinical screening requirements.

In the PI classification task, the high threshold produced a marked impact on performance and revealed differences in the robustness of the algorithms. The most significant changes occurred for Random Forest and SVM. Random Forest previously achieved the highest recall of 0.7646 under the default threshold, but its recall dropped sharply to 0.1418 under the 80 percent threshold, and its F1-score fell to 0.2376. The recall of SVM also dropped to 0.1506. This suggests that although these models can classify correctly, their predicted probabilities are often concentrated between 0.5 and 0.8 and they lack the ability to provide highly confident predictions. In contrast, XGBoost and LightGBM exhibited strong resilience. XGBoost achieved the highest F1-score of 0.6348 and the highest recall of 0.5266 under this task, with LightGBM performing slightly behind. This indicates that XGBoost and LightGBM not only classify accurately but also provide better calibration of prediction confidence, allowing them to more confidently identify PI samples.

In summary, in scenarios requiring high confidence decisions, LightGBM and XGBoost hold substantial advantages over Random Forest and traditional machine learning models. This advantage is especially pronounced in the PI task, where feature relationships are more ambiguous and these models are better suited to supporting diagnostic decision making.

### 3.3 External validation results on disease classification

**FIGURE 6:**
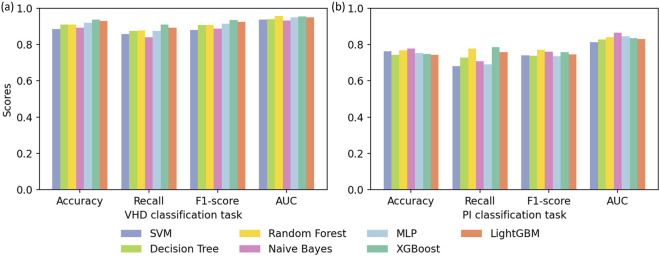
Results of external validation experiments for **(a)** the VHD classification task and **(b)** the PI classification task.

To assess the generalization performance of the model on external data, we designed an external validation experiment. We sampled the training set from patient data from 8 hospitals and the validation set from data from another 2 hospitals. The ratio of the training set to the validation set was about 9:1, and the ratio of positive and negative samples was 1:1. Each model was evaluated 10 times, and the results are summarized in Figure 6.

Experimental results indicate that all baseline models demonstrated strong generalization capability on the external dataset. Compared with the tenfold cross validation results reported in Figure 3, the performance metrics of each model improved to varying degrees in the external validation experiments, which indicates that the trained models did not suffer from overfitting and adapted well to distributional differences across centers. Overall, ensemble learning models represented by XGBoost, LightGBM and Random Forest remained the most competitive across both tasks, showing robustness and stability when handling complex medical data. The consistent performance of these models on the external validation set further supports the feasibility of deploying the classification system in clinical practice.

### 3.4 Influence of hyperparameters

We tested the number of Decision Trees, maximum depth, and minimum number of samples required for leaf nodes of the Random Forest hyperparameters on the PI classification by adjusting the size of each parameter to test the robustness of the model.

**FIGURE 7:**
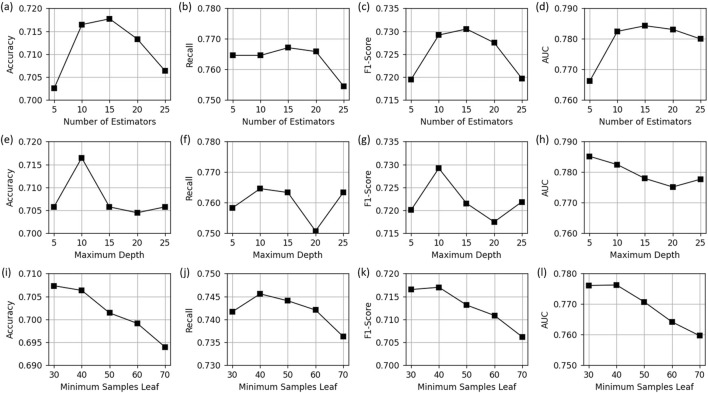
Impact of hyperparameters on the performance of Random Forest model. **(a–d)** shows the impact of the number of estimators, **(e–h)** shows the impact of the maximum depth, and **(j–l)** shows the impact of the minimum samples leaf.

The number of Decision Trees has a great impact on the performance of the model, so we set different numbers for experiments (as shown in Figures 7a–d). It can be seen that the model performance is best when 15 Decision Trees are integrated. Appropriately increasing the number of Decision Trees can usually improve the performance of the model because it can fit the data more fully and reduce the deviation. However, when it increases to a certain extent, the performance may decrease due to overfitting, and computing resources will be wasted.

We studied the impact of the maximum depth of the Decision Tree on the model performance (as shown in Figures 7e–h). Specifically, the accuracy, F1-score, and AUC reach the highest when the maximum depth is 10, and the recall rate is relatively stable when the depth changes, and the range of variation is minimal. Too small or too large depth will cause the model to underfit or overfit, affecting the model performance.

We changed the minimum number of samples required for leaf nodes from 30 to 70 to study its impact on model performance (see Figures 7i–l). Smaller values give the model more degrees of freedom to segment data and better capture complex feature relationships. Larger values can enhance model regularization and reduce the risk of model overfitting. In this classification, setting the minimum number of samples required for leaf nodes to 40 can achieve the best performance.

### 3.5 Model interpretation analysis

**FIGURE 8:**
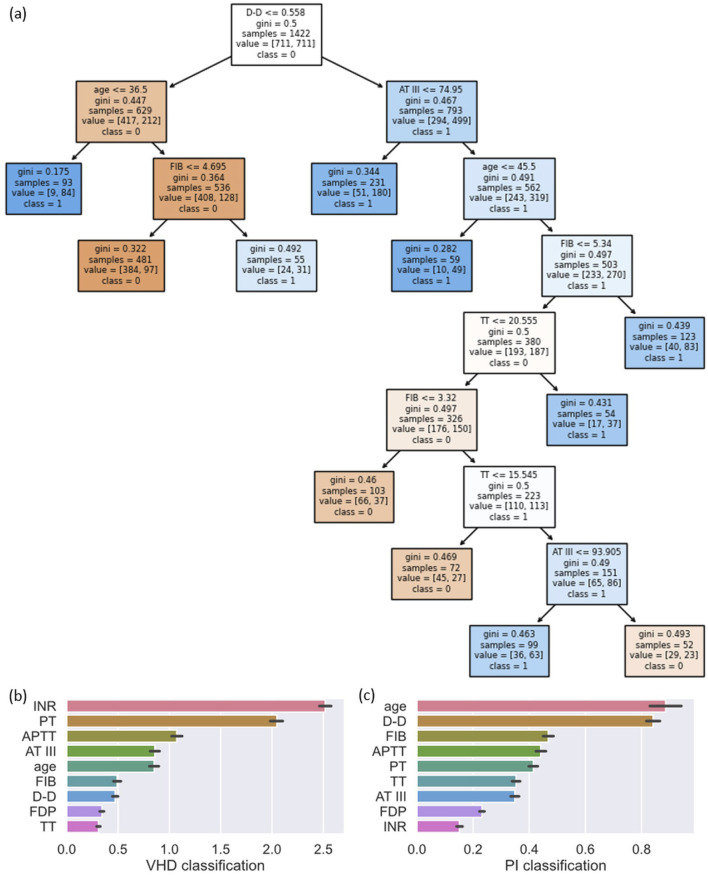
Feature visualization of Decision Tree for PI classification **(a)** and feature importance for VHD and PI classification tasks **(b,c)**. The parameters in the Decision Tree nodes are interpreted as follows: the inequality represents the splitting feature and its threshold; gini represents the Gini impurity; samples represents the number of samples reaching the node; value represents the distribution of samples per class; and class represents the predicted majority class.

First, we explain the classification mechanism of the model by directly analyzing the structure of the model. Since the tree structure has good interpretability, we use the structure diagram of the Decision Tree for analysis. The Decision Tree model trained in the PI classification is shown in Figure 8a. D-D is the root node, indicating that it has the largest weight in sample classification. Age and AT III appear in nodes close to the root, showing their importance for early classification. FIB appears in multiple nodes, indicating that it has a strong ability to distinguish between the two types of samples.

In order to further explore the contribution ratio of each feature in the model to the results, we used the SHAP library to analyze the LightGBM model trained in the two classification tasks, and the results are shown in Figures 8b,c.

In the VHD classification, INR has the greatest contribution to model prediction, followed by PT. This is because patients with VHD need to take long-term anticoagulant drugs to prevent valve thrombosis, resulting in significantly different indicators such as INR and PT used to evaluate the anticoagulant effect compared with other diseases. In addition, abnormal blood flow in VHD and long-term vascular endothelial stimulation can lead to active extrinsic coagulation pathway, and PT and INR are core indicators of the extrinsic coagulation pathway. The combined effect of these factors makes PT and INR significantly higher in patients with VHD than in patients with other diseases.

In the PI classification, age is the most important contributing feature, followed by D-D and FIB, with APTT also showing a high level of contribution. These findings are consistent with the analysis of the Decision Tree models and align closely with clinical and pathological characteristics. Older adults have relatively weaker immune function and reduced respiratory defense mechanisms, which increases their susceptibility to PI and makes age a key feature for distinguishing infection cases. D-D and FIB ranked second and third, which aligns with known pathophysiological mechanisms, as severe infections often trigger systemic inflammatory responses and abnormalities in the coagulation and fibrinolytic systems. This leads to marked changes in the levels of FIB, which is an acute phase reactant protein, and D-D, which reflects secondary hyperfibrinolysis. Indicators such as APTT also played an important role in assisting the differentiation of infection samples.

The analysis of individual patient characteristics further validates the SHAP results. As shown in Table 3, the correctly predicted Patient 1 exhibits significantly elevated INR and PT, aligning with the high feature importance of these indicators. In contrast, the false negative (Patient 3) shows normal INR and PT despite having VHD, leading to a missed detection due to the absence of typical features. Conversely, the false positive (Patient 4) presents markedly elevated INR and PT due to non-VHD clinical factors, inducing a misclassification. These local case analyses further demonstrate the predominant influence of coagulation indicators on model decision-making and highlight the necessity of integrating broader clinical context to mitigate such misclassifications in future research.

**TABLE 3:** Detailed clinical indicators and prediction results for representative VHD patients. Label indicates the ground-truth clinical diagnosis (1 for positive, 0 for negative), and Pred. denotes the model’s predicted probability.

Patient	INR	PT	APTT	AT III	Age	FIB	D-D	FDP	TT	Label	Pred	Result
1	3.09	34.50	44.34	117.12	57	3.88	0.27	0.74	15.30	1	>0.99	Correct
2	0.96	11.31	27.34	88.91	70	3.12	0.19	0.74	15.01	0	<0.01	Correct
3	1.07	12.35	28.57	82.03	77	2.33	6.29	18.09	39.61	1	<0.01	Incorrect
4	2.79	31.30	42.15	83.30	69	2.87	0.83	2.41	18.38	0	>0.99	Incorrect

## 4 Discussion

This study examined the classification tasks of VHD and PI by analyzing clinical data from patients, and compared the performance of seven machine learning models. The results showed that the LightGBM model performed best in both tasks. With its ensemble learning characteristics, it effectively processes high-dimensional data and captures complex, nonlinear feature relationships. In addition, external validation experiments confirmed the model’s generalization, while data visualization, hyperparameter experiments, and SHAP analysis validated its effectiveness and interpretability.

Despite these meaningful results, several limitations must be addressed to enhance the clinical utility of our findings. First, while all 11 hospitals utilized the CX9000 automatic coagulation analyzer, potential inter-center variations in assay standardization and laboratory protocols remain a concern. Although our external validation showed strong generalization, future studies should implement more rigorous cross-site calibration or domain adaptation techniques to minimize center-specific bias.

Second, a critical limitation involves confounding factors, particularly patient medication history. As highlighted by our SHAP analysis, INR and PT were the most significant features for VHD classification. Given that patients with VHD frequently undergo long-term anticoagulant therapy to prevent thrombosis, these laboratory abnormalities likely reflect the pharmacodynamic effect of medication rather than solely the biological progression of the disease. In this study, medication records were not explicitly integrated into the model training as independent features. Future iterations of this model should incorporate detailed medication history to differentiate between therapy-induced changes and primary pathological signatures, thereby providing a more nuanced diagnostic tool.

Furthermore, this study focused on classic machine learning and boosting architectures. However, the potential of Transformer-based architectures in disease classification warrants exploration. Unlike boosting models, Transformers utilize self-attention mechanisms that can capture higher-order, global interactions between tabular features. Studies on tabular Transformers Huang et al. (2020); Gorishniy et al. (2021) suggest that they may offer superior performance in handling complex, multimodal medical data, especially as dataset sizes increase.

In conclusion, by explaining the importance of features for individual predictions and overall predictions, we revealed key indicators affecting disease classification. While the current study provides data-driven decision support for doctors, future work should expand to multi-classification tasks, integrate multimodal features like imaging or genetic information, and explore deep learning architectures to further optimize clinical transparency and credibility.

## 5 Conclusion

This study focuses on the analysis and classification of patient clinical data. After analyzing the patient characteristic indicators, two binary classification tasks were designed for VHD and PI. The experimental results show that the machine learning method based on LightGBM can not only effectively improve the performance of disease classification, but also help identify key indicators that affect classification through explanatory analysis, providing a new technical path for medical auxiliary diagnosis. In the future, we can explore more complex multi-classification and multi-label tasks, and combine deep learning models to process high-dimensional medical data more efficiently. At the same time, the data scale can be expanded and multimodal features such as imaging and genetic information can be introduced to improve the generalization ability of the model. In addition, the interpretability of the model is further optimized to make it more transparent and credible in medical practice.

## Data Availability

The raw data supporting the conclusions of this article will be made available by the authors, without undue reservation.
